# Demonstration of a WNT5A-IL-6 positive feedback loop in melanoma cells: Dual interference of this loop more effectively impairs melanoma cell invasion

**DOI:** 10.18632/oncotarget.9332

**Published:** 2016-05-12

**Authors:** Rickard Linnskog, Purusottam Mohapatra, Farnaz Moradi, Chandra Prakash Prasad, Tommy Andersson

**Affiliations:** ^1^ Cell and Experimental Pathology, Department of Translational Medicine, Lund University, Clinical Research Centre, Skåne University Hospital, SE-20502 Malmö, Sweden

**Keywords:** WNT5A, interleukin-6, anti-IL-6-Ab, Box5, melanoma invasion

## Abstract

Increased expression and signalling of WNT5A and interleukin-6 (IL-6) have both been shown to promote melanoma progression. Here, we investigated the proposed existence of a WNT5A-IL-6 positive feedback loop that drives melanoma migration and invasion. First, the HOPP algorithm revealed that the invasive phenotype of cultured melanoma cells was significantly correlated with increased expression of *WNT5A* or IL-6. In three invasive melanoma cell lines, endogenous WNT5A protein expression was related to IL-6 protein secretion. Knockdown with anti-IL-6 siRNAs or treating WM852 melanoma cells with a neutralising anti-IL-6 antibody reduced WNT5A protein expression. Conversely, the silencing of WNT5A expression by WNT5A siRNAs or treating WM852 melanoma cells with Box5 (a WNT5A antagonist) significantly reduced IL-6 secretion. Interestingly, these effects occurred at the protein level but not at the transcriptional levels. Functionally, we demonstrated that combined siRNA knockdown of WNT5A and IL-6 expression or the simultaneous inhibition of WNT5A and IL-6 signalling inhibited melanoma cell invasion more effectively than suppressing each factor individually. Together, our results demonstrate that WNT5A and IL-6 are connected through a positive feedback loop in melanoma cells and that the combined targeting of both molecules could serve as an effective therapeutic means to reduce melanoma metastasis.

## INTRODUCTION

Deregulation of *WNT5A* is often linked to the development and progression of various cancers [1]. While the loss of WNT5A expression is correlated with poor prognosis in breast [2] and colorectal cancer [3], the opposite trend was observed for cutaneous melanoma [4]. Increased WNT5A expression is associated with a higher invasive and metastatic potential of melanoma cells [5, 6]. Similar to WNT5A, the pro-inflammatory cytokine IL-6 promotes melanoma cell invasion, and its increased expression is correlated with reduced overall patient survival [7–10]. Two recent studies have demonstrated a link between IL-6 secretion and WNT5A expression in melanoma cells [11, 12], suggesting that the combined therapeutic interference with this link might be beneficial for preventing disease progression and metastatic spread.

WNT5A is a lipid-modified secreted glycoprotein that is regarded as a non-canonical WNT ligand, which means that it elicits the activation of β-catenin-independent WNT signalling pathways [13]. In turn, these pathways can be subdivided depending on the major downstream signalling molecule involved (e.g., Ca^2+^, JNK and small GTPases such as Rho, Rac and Cdc42), and their selective activation is largely dictated by the cell surface context of different non-canonical WNT receptors [14, 15]. Certain members of the Frizzled family of GPCRs and tyrosine kinase receptors such as ROR2 and RYK have been demonstrated to mediate WNT5A-induced β-catenin-independent signalling [1, 16, 17]. In melanoma, many of these pathways have been directly shown to participate in WNT5A-driven cell migration and invasion [5, 18, 19]. Considering all of these factors, we have developed a WNT5A-derived antagonistic peptide that could be used to inhibit WNT5A signalling and subsequently reduce melanoma cell invasion [20].

Apart from WNT5A, there are other regulators of melanoma cell invasion that promote metastasis; IL-6 is one of these regulators. In cutaneous melanoma, IL-6 expression is detectable at the early nevi stage, and its level dramatically increases as the tumour invades deeper into the underlying dermis [10]. Similar to the IL-6 level, the expression of the IL-6 receptor (IL-6R) also increases with melanoma progression, indicating an autocrine or paracrine function for IL-6 during melanoma progression [10]. In the classical signalling pathway, IL-6 acts by binding to IL-6R, a receptor complex of IL-6Rα and glycoprotein 130 (gp130) receptors. IL-6 binding to IL-6R induces JAK-mediated phosphorylation of several tyrosine receptor motifs within the cytosolic domain of gp130, which activates the transcription factors of the STAT-family and also mediates the activation of RAS/RAF/MEK/MAPK and PI3K/AKT-signalling [21]. In agreement to these classical pathways, we have recently shown that IL-6 can induce p38α-MAPK activation in melanoma cells. More importantly, we demonstrated that the IL-6-induced p38α-MAPK activation promoted melanoma cell migration and invasion through increased WNT5A expression [12].

The aim of the current study was to explore the existence of a WNT5A-IL-6 positive feedback loop in malignant melanoma cells and to investigate whether dual interference with this loop would be a more effective therapeutic means to obstruct melanoma cell migration and invasion.

## RESULTS

### Elevated WNT5A and IL-6 expressions in invasive melanoma

**Figure 1:**
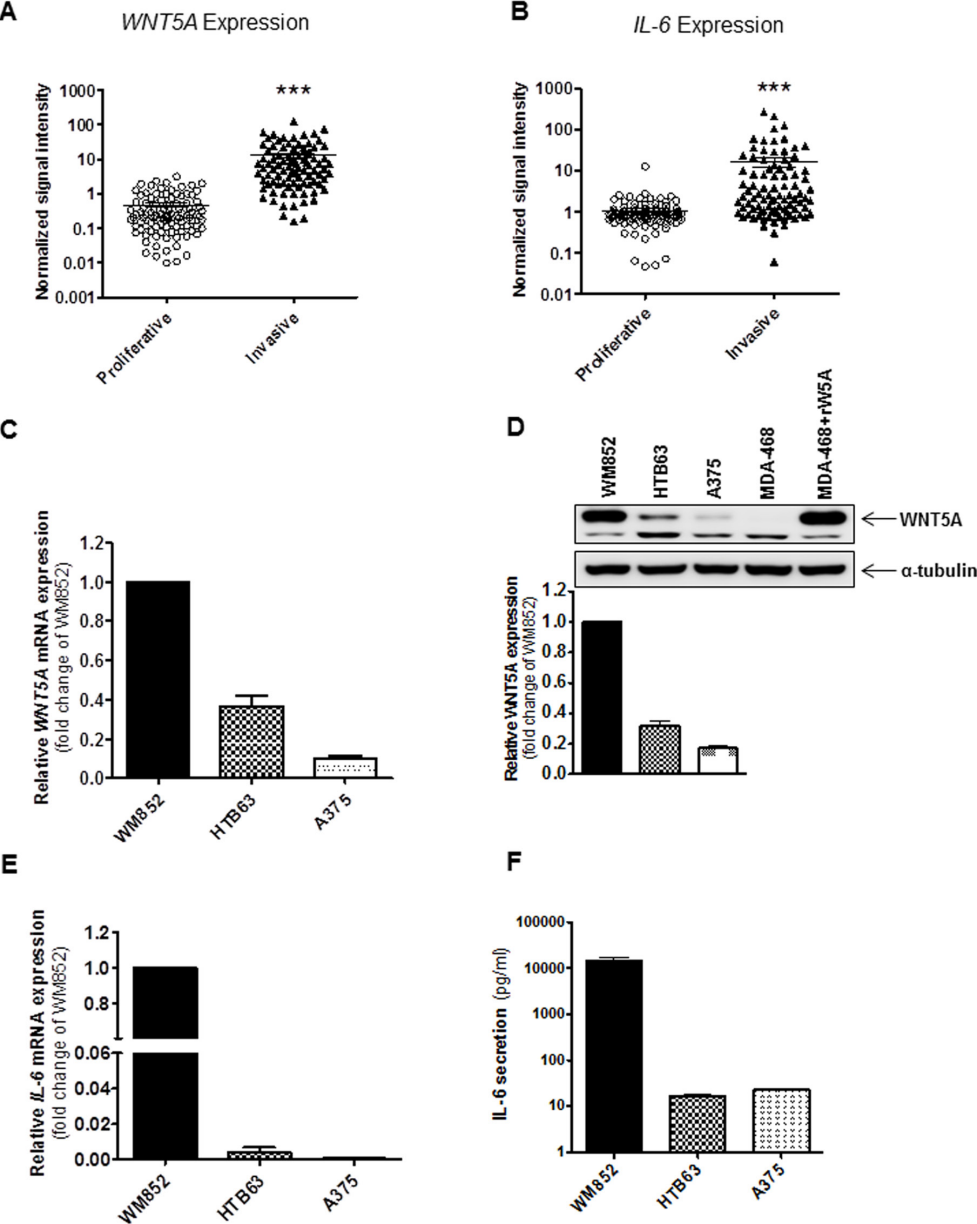
Increased expression of WNT5A and IL-6 is associated with an invasive melanoma cell phenotype (**A**, **B**) The panels show the gene expression of *WNT5A* and *IL-6* in multiple microarray data sets of melanoma cell lines classified by the Heuristic Online Phenotype Prediction (HOPP) algorithm as proliferative versus invasive. (**C**) QPCR analyses of endogenous *WNT5A* mRNA expression levels in human WM852, HTB63, and A375 cells. Samples were normalised against *TATA-binding protein (TBP)* mRNA expression, and the results are presented relative to the *WNT5A* mRNA expression of WM852 cells (*n* = 5). (**D**) Western blot analysis showing the levels of endogenous WNT5A protein expression in the human melanoma cell lines WM852, HTB63, and A375. α-tubulin was used as a loading control. A representative blot from four separate experiments is shown. The lower panel shows densitometric analyses of WNT5A protein expression normalised against α-tubulin; the results are presented relative to WNT5A protein expression of WM852 cells (*n* = 4). (**E**) QPCR analysis of endogenous *IL-6* mRNA expression levels in human WM852, HTB63, and A375 cells. Samples were normalised against *TATA-binding protein (TBP)* mRNA expression; the results are presented relative to the *IL-6* mRNA expression of WM852 cells (*n* = 5). (**F**) ELISA analysis showing the quantification of endogenous IL-6 protein secretion in human WM852, HTB63, and A375 cells (*n* = 5). The results are presented as the means and S.E.Ms; ***p < 0.001.

To test our hypothesis that WNT5A and IL-6 could co-operate to accelerate melanoma metastasis, we first analysed whether their gene expression levels correlated with the invasive potential of melanoma cell lines. This investigation was possible due to the Heuristic Online Phenotype Prediction (HOPP) algorithm developed by Hoek and colleagues. The algorithm phenotypically stratifies publicly available microarray data sets to classify individual melanoma cell lines as either proliferative or invasive [22]. As previously demonstrated [12], extracted data revealed that significantly increased mRNA expression of *WNT5A* (Figure 1A) is associated with an invasive phenotype signature of melanoma cells. Interestingly, the same association was discovered for the mRNA expression of *IL-6* (Figure 1B). We also performed a correlation analysis between the two ligands on an individual cell line basis. However, we found only a poor correlation (Pearson correlation = 0.194) between *WNT5A* and *IL-6* mRNA expression (data not shown) in the invasive melanoma cell lines. In proliferative cell lines, our analyses revealed a similar weak correlation (Pearson correlation = 0.254) between *WNT5A* and *IL-6* mRNA expression (data not shown). We also analysed the co-expression of *WNT5A* and *IL-6* mRNA in different melanoma tumour tissue data sets by using the TCGA melanoma data set (www.cancergenome.nih.gov) and Oncomine melanoma data sets (www.oncomine.org). Our results showed either poor or no correlation between *WNT5A* and *IL-6* mRNA expression in patient-derived melanoma samples (data not shown).

Next, we selected three melanoma cell lines—WM852, HTB63 and A375—that are phenotypically classified as invasive by the HOPP algorithm (Figure S1), and compared their levels of *WNT5A* and *IL-6* mRNA/protein expression. In good agreement with our previous report [12], all three cell lines expressed *WNT5A* mRNA and protein, although WM852 cells displayed a significantly higher level of expression compared to HTB63 and A375 cells (Figure 1C and 1D). Similarly, QPCR and ELISA-based analysis of the three cell lines and their conditioned cell culture media demonstrated that the level of *IL-6* mRNA and IL-6 secretion was several magnitudes higher in WM852 cells compared to HTB63 and A375 cells (Figure 1E and 1F). However, because we also observed a difference in WNT5A protein expression between HTB63 and A375 cells (Figure 1D), we anticipated a similar difference when comparing their levels of IL-6 secretion. The reason why no difference was detected (Figure 1F) is presently unknown. Interestingly, as presented in Figure 1C and 1E, we did not observe any correlation between *WNT5A* and *IL-6* mRNA expression. Furthermore, the quantitative PCR analysis revealed a significantly higher transcriptional level of the IL-6 receptor (IL-6R) in HTB63 cells compared to WM852 and A375 cells (Figure S2). Together, these analyses and our cell line data suggest that *WNT5A* and *IL-6* mRNA expression is poorly correlated in melanoma cells, although their increased expression is characteristic of an invasive melanoma cell phenotype. The three melanoma cell lines (WM852, HTB63 and A375) studied expressed different levels of WNT5A protein and secreted different amounts of IL-6 (Figure 1D and 1F). However, due to the limited number of cell lines used in our study, their WNT5A protein levels and IL-6 secretion could not be correlated. Therefore, we investigated their possible relationship by different means.

### IL-6 increases the expression and release of WNT5A in WM852 cells

**Figure 2:**
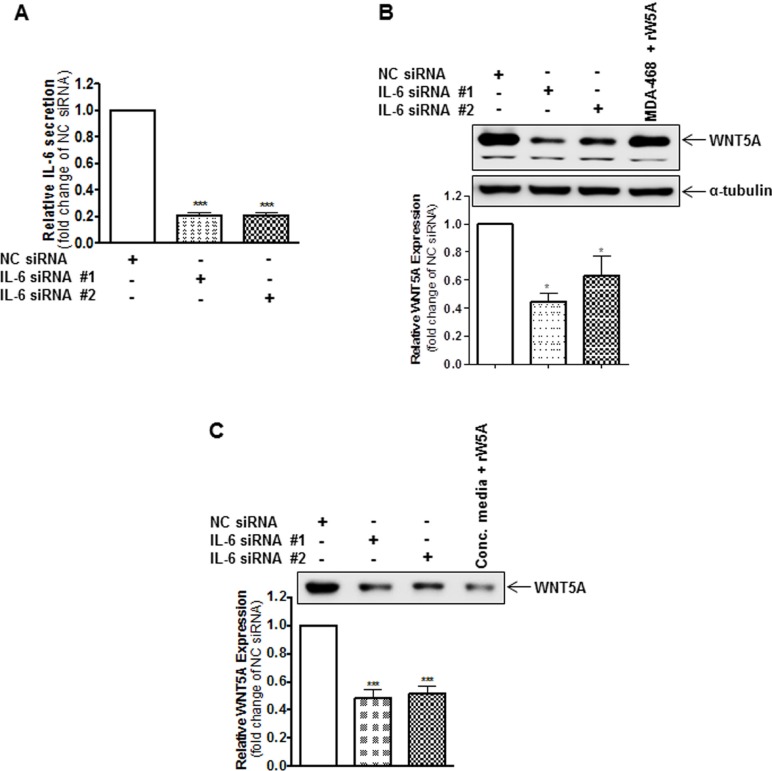
IL-6 knockdown reduces the endogenous protein expression of WNT5A in WM852 cells (**A**) ELISA analysis showing siRNA-based silencing of endogenous IL-6 in WM852 cells. Cells were transfected with either negative control siRNA (NC siRNA; 50 nM), anti-IL-6-siRNA #1 (IL-6 siRNA #1; 50 nM) or anti-IL-6-siRNA #2 (IL-6 siRNA #2; 50 nM) and incubated for 72 h. The results are presented relative to the IL-6 secretion of NC siRNA-transfected cells (*n* = 4). (**B**) Western blot analysis of WNT5A expression in WM852 cells treated as described in (A). Densitometric analyses were performed, and the data were then normalised to α-tubulin, which was used as a loading control. The results are presented relative to the WNT5A expression of NC siRNA-transfected cells (*n* = 4). (**C**) Western blot analysis of WNT5A release from WM852 cells treated as described in (A). A representative blot from four separate experiments is shown. The results are presented as the means and S.E.Ms; *p < 0.05; ***p < 0.001.

**Figure 3:**
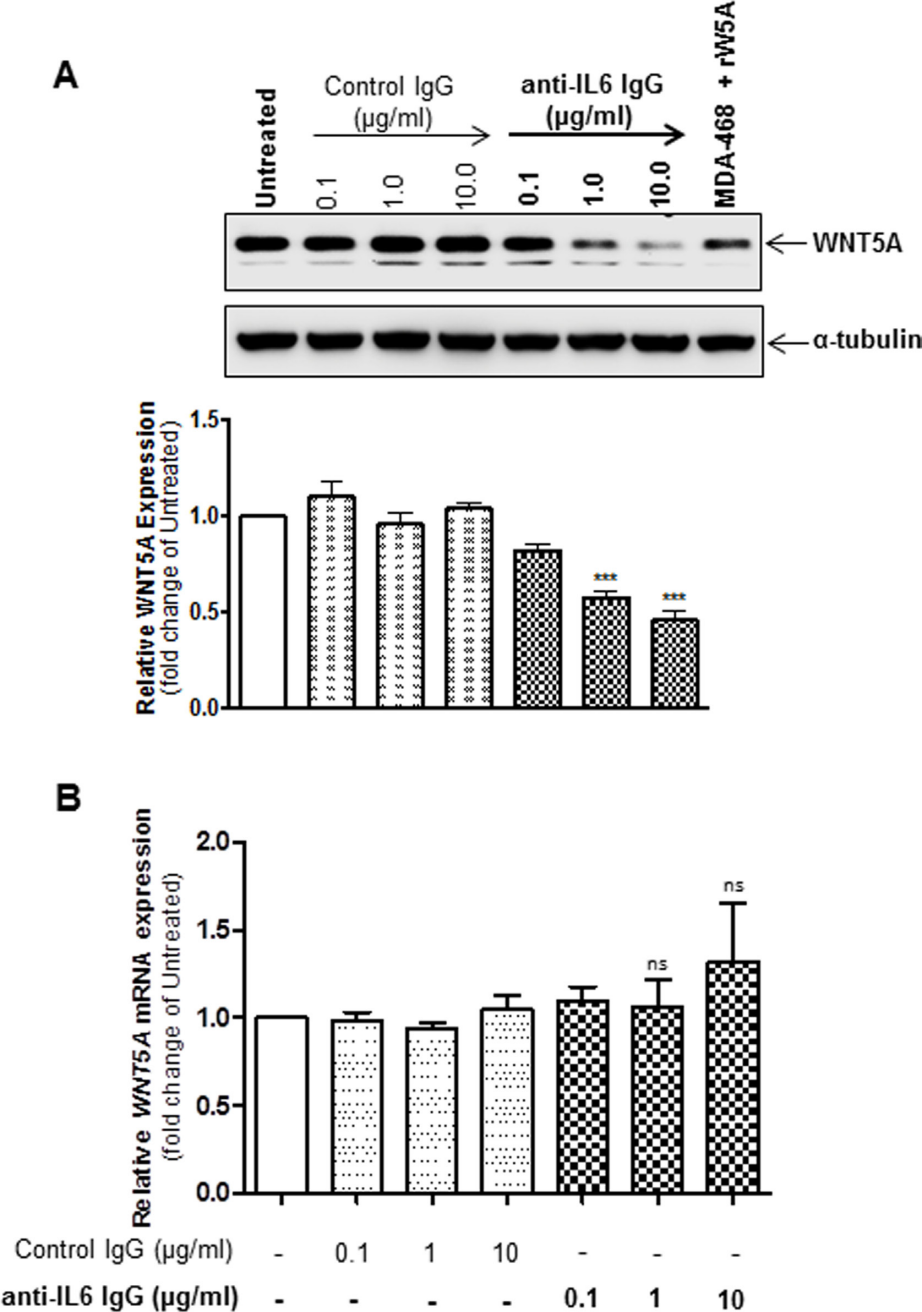
Inhibition of IL-6 signalling reduces WNT5A proteins expression but not *WNT5A* mRNA expression in WM852 cells (**A**) Western blot analysis of WNT5A expression in WM852 cells treated with the indicated concentrations of a neutralising anti-IL-6 antibody or equal concentrations of an IgG1 isotype control antibody. Densitometric analyses were performed, and the data were then normalised to α-tubulin used as a loading control. The results are presented relative to the WNT5A expression of untreated WM852 cells (*n* = 5). (**B**) QPCR analyses of endogenous *WNT5A* mRNA expression levels in human WM852 treated with the indicated concentrations of a neutralising IL-6 antibody or equal concentrations of an IgG1 isotype control antibody. Samples were normalised against *TATA-binding protein (TBP)* mRNA expression, and the results are presented relative to the *WNT5A* mRNA expression of untreated cells (n = 5). The results are presented as the means and S.E.Ms; ***p < 0.001.

We previously demonstrated that treatment with recombinant IL-6 (rIL-6) significantly increases the expression of WNT5A in HTB63 and A375 cells, but it had no effect on WNT5A expression in WM852 cells [12]. Based on the observations made by Terai *et al.*, in which rIL-6-induced IL-10 secretion was much more pronounced in melanoma cells with low compared to high endogenous IL-6 secretion [23], our results might have been due to a high level of endogenous IL-6 secretion in WM852 cells (Figure 1F). Consequently, to investigate whether WNT5A expression is regulated by IL-6 in WM852 cells, we explored how knockdown of IL-6 by two independent siRNAs affected WNT5A expression. Compared to NC siRNA-transfected cells, the two different IL-6 siRNAs significantly reduced IL-6 secretion 72 h post-transfection (Figure 2A). Interestingly, the reduced IL-6 secretion was concomitantly associated with significantly lowered WNT5A expression (Figure 2B) and release (Figure 2C), thus indicating that their high level of IL-6 secretion maintained the relatively high WNT5A expression in WM852 cells. To validate these findings, we studied whether the recombinant-IL-6 stimulation of IL-6 siRNA-depleted WM852 cells could regain WNT5A release after 48 h. In agreement with our assumption of a WNT5A-IL-6 positive feedback loop, we observed an increased release of WNT5A in the concentrated medium of recombinant-IL-6-stimulated IL-6-silenced WM852 cells after 48 h (Figure S3). To confirm these results, we treated WM852 cells for 24 h with increasing concentrations of a monoclonal anti-IL-6 antibody to neutralise endogenous IL-6 signalling. In accordance with our previous results, increasing concentrations of the neutralising IL-6 antibody reduced WNT5A expression in a dose-dependent manner, whereas equal concentrations of an IgG_1_ isotype control antibody had no effect (Figure 3A). Cell viability studies did not reveal any significant effect of the human anti-IL-6 antibody on WM852 cell viability (Figure S4). To determine whether the IL-6-induced effect on WNT5A expression occurs at the transcriptional or translational level, the *WNT5A* mRNA level was also evaluated in WM852 cells after monoclonal anti-IL-6 antibody treatment. Interestingly, there was no significant reduction in the *WNT5A* mRNA level after treating the cells with increasing doses of the IL-6 neutralising antibody (Figure 3B). These results confirm that IL-6 positively regulates WNT5A expression in WM852 melanoma cells, and that this regulation occurs at the translational level.

### WNT5A increases the release of IL-6 in WM852 cells

**Figure 4:**
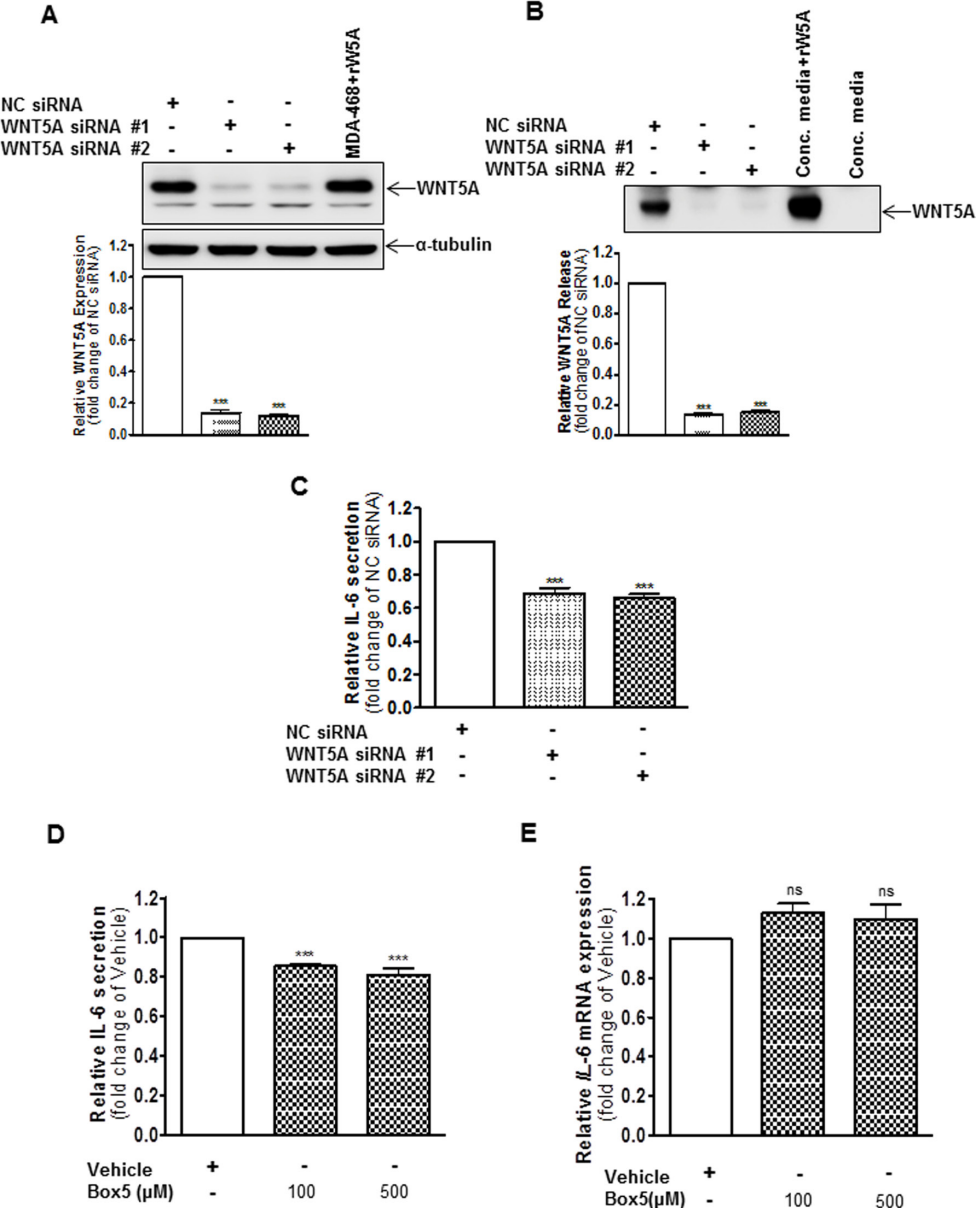
WNT5A increases IL-6 secretion from WM852 cells (**A**) Western blot analysis showing siRNA silencing of endogenous WNT5A expression in the human melanoma cell line WM852. Cells were transfected with either negative control siRNA (NC siRNA; 100 nM), anti-*WNT5A*-siRNA #1 (WNT5A siRNA #1; 100 nM) or anti-*WNT5A*-siRNA #2 (WNT5A siRNA #2; 100 nM) and were incubated for 72 h. Densitometric analyses were performed, and the data were then normalised to α-tubulin, which was used as a loading control. The results are presented relative to the WNT5A expression of NC siRNA-transfected cells (*n* = 4). (**B**) Western blot analysis of WNT5A release from WM852 cells treated as described in (A). A representative blot from four separate experiments is shown. (**C**) ELISA analysis of IL-6 secretion from WM852 cells treated as described in (A). The results are presented as relative to IL-6 secretion of NC siRNA-transfected cells (*n* = 4). (**D**) ELISA analysis of IL-6 secretion from WM852 cells treated with the indicated concentrations of Box5 or vehicle control (NaHCO_3_ buffer, pH ~7). The results are presented relative to IL-6 secretion of vehicle-treated cells (*n* = 4). (**E**) QPCR analyses of *IL-6* mRNA expression levels in human WM852 treated with the indicated concentrations of Box5 or vehicle control (NaHCO_3_ buffer, pH ~7). Samples were normalised against *TATA-binding protein (TBP)* mRNA expression, and the results are presented relative to the *IL-6* mRNA expression of vehicle-treated cells (*n* = 5). The results are presented as the means and S.E.Ms; ***p < 0.001.

Having ascertained that IL-6 increases WNT5A expression in WM852 cells, we next investigated whether a reciprocal relationship exists between the two factors. Transient transfection (72 h) using two independent WNT5A-siRNAs resulted in significantly reduced WNT5A expression (Figure 4A) and release (Figure 4B) compared to NC siRNA-transfected cells. Notably, when the conditioned media from the same cells was analysed for the content of IL-6, both of the WNT5A-siRNAs caused a statistically significant reduction in IL-6 release compared to the NC siRNA-transfected cells (Figure 4C), indicating that WNT5A increases IL-6 release in WM852 cells. These results are in good agreement with previously published observations in which siRNA silencing of WNT5A reduced the exosomal release of IL-6 in HTB63 cells [11]. To confirm our results and also take a more therapeutic approach, we then treated WM852 cells for 24 h with 100 or 500 μM of the WNT5A-derived antagonistic peptide ‘Box5’ to inhibit endogenous WNT5A signalling. Although no significant difference was observed between the two different concentrations of Box5, they both mimicked the results obtained through siRNA-silenced WNT5A expression, lowering the IL-6 release compared to that in vehicle-treated cells (Figure 4D). To investigate whether the WNT5A-induced IL-6 secretion is regulated at the transcriptional level, the *IL-6* mRNA level was evaluated by QPCR in WM852 cells after Box5 (100 and 500 μM) treatment. Interestingly, there was no reduction in the *IL-6* mRNA expression which favours the non-transcriptional regulation of IL-6 after antagonising the effect of WNT5A by Box5 treatment (Figure 4E). There was no significant inhibition of WM852 cell viability after treatment with 100 μM of Box5. However, limited toxicity was observed after treatment with 500 μM of Box5, mainly due to the increased DMSO concentration (Figure S5). These data demonstrate that WNT5A increases IL-6 secretion in WM852 cells. Together, our observations provide evidence for the existence of an endogenous WNT5A-IL-6 positive feedback loop mechanism that facilitates the increased expression of these two pro-metastatic factors in WM852 melanoma cells.

### Dual interference of the WNT5A-IL-6 positive feedback loop more effectively impairs melanoma cell migration and invasion

Several studies have highlighted the ability of either IL-6 or WNT5A to promote melanoma cell motility [5, 7, 9, 18–20]. As these findings and our present data demonstrate a WNT5A-IL-6 positive feedback loop in WM852 melanoma cells, we investigated whether targeting both these factors simultaneously could be a more efficient way to reduce or inhibit melanoma cell migration and invasion.

**Figure 5:**
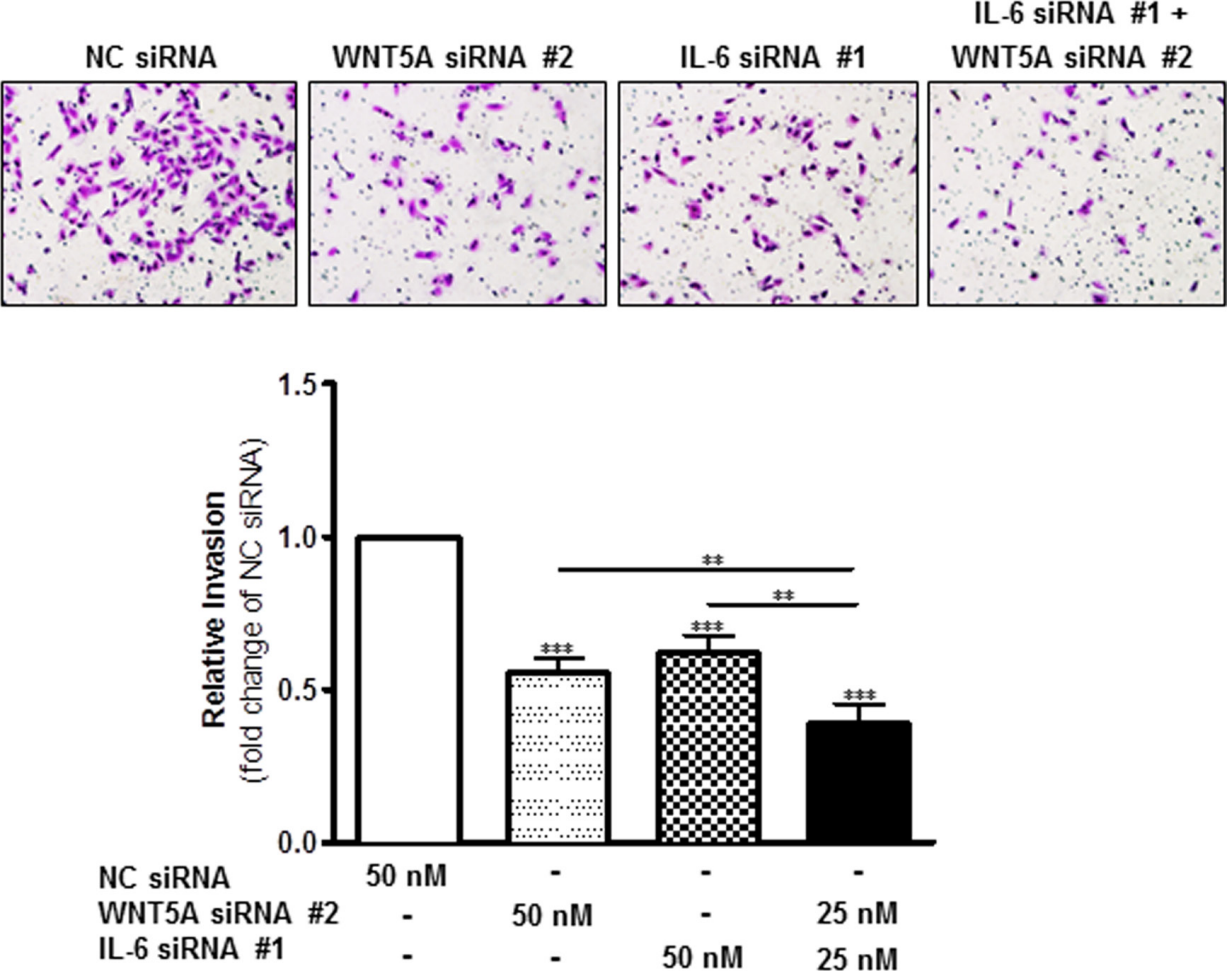
Combined knockdown of WNT5A and IL-6 protein expression more effectively inhibits WM852 cell invasion In a Matrigel-based invasion assay system, the human melanoma cell line WM852 was transiently transfected for 72 h with either negative control siRNA (NC siRNA; 50 nM), anti-WNT5A-siRNA #2 (WNT5A siRNA #2; 50 nM), anti-IL-6-siRNA #1 (IL-6 siRNA #1; 50 nM) or anti-WNT5A-siRNA #2 and anti-IL-6-siRNA #1 (25 nM each). The invasion assays were performed during the last 24 h of each transient transfection and analysed as described in the Materials and Methods section. The results are presented as the relative invasion of NC siRNA transfected cells (*n* = 4) and are presented as the means and S.E.Ms; *p < 0.05; **p < 0.01; ***p < 0.001.

**Figure 6:**
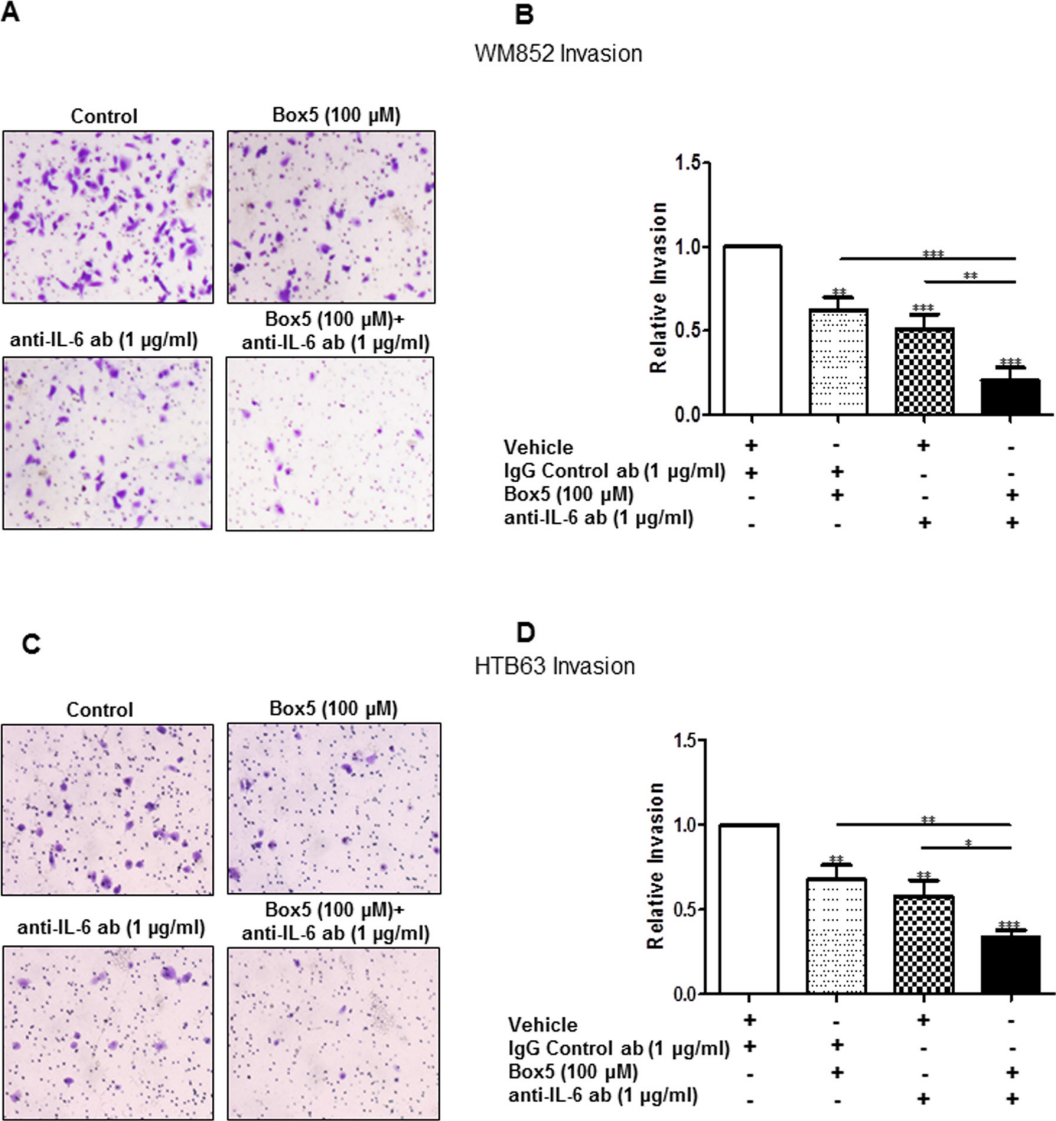
Combined inhibition of WNT5A and IL-6 signalling with Box5 and an anti-IL-6 antibody more effectively impairs WM852 and HTB63 cell invasion Matrigel invasion of WM852 (**A** and **B**) and HTB63 (**C** and **D**) cells treated with either Box5 or neutralising anti-IL-6 antibody alone and their combination was evaluated as described under the Materials and Methods section. Proper controls (DMSO for Box5 treatment or IgG_1_ isotype antibody for neutralising anti-IL-6-antibody treatment) were used to compare the treatment effect on the cell invasions. The invasion assays (*n* = 5) were performed over 24 h and analysed as described in the Materials and Methods section. The results are presented as the means and S.E.Ms; *p < 0.05; **p < 0.01; ***p < 0.001.

To investigate this phenomenon, we first transiently transfected WM852 cells for 72 h with anti-WNT5A siRNA #2 alone, anti-IL-6 siRNA #1 alone, or a combination of both, and we assessed their invasive capability relative to NC siRNA-transfected cells. Strikingly, although siRNA silencing of either WNT5A or IL-6 (Figure 5) alone reduced WM852 cell invasion by approximately 40%, the combined silenced expression of both WNT5A and IL-6 further reduced WM852 cell invasion to approximately 60% (Figure 5). Taking a more feasible therapeutic approach, we treated two different melanoma cell lines (WM852 and HTB63) with Box5 (100 μM) and/or a neutralising anti-IL-6 antibody (1 μg/ml) and checked their migration/invasion. In both cell lines, we observed that combined blockage of WNT5A and IL-6 signalling more efficiently impaired cell migration/invasion (Figures 6 and S6), similar to the results obtained with combined siRNA transfection (Figure 5). We did not observe any significant effects on WM852 cell viability after Box5 (100 μM) and/or anti-IL-6 antibody (1 μg/ml) treatment (Figure S7). Our data clearly indicate that WNT5A and IL-6 co-operate to facilitate melanoma cell migration and invasion and that the combined therapeutic inhibition of both WNT5A and IL-6 signalling stands out as an effective treatment strategy to prevent melanoma dissemination.

## DISCUSSION

In a study on rheumatoid arthritis (RA), Sen et al. showed that WNT5A could induce the expression of the pro-inflammatory cytokine IL-6 in fibroblast-like synoviocytes (FLS) and the inhibition of WNT5A either with antisense or a dominant negative vector, reducing IL-6 levels in FLS obtained from patients with RA [24]. Moreover, it has been demonstrated that WNT5A can positively regulate the exosomal release of IL-6 [11], and we have shown that stimulation with exogenous IL-6 can induce WNT5A expression in malignant melanoma cells [12]. Taking into account these findings, we set out to investigate whether WNT5A can regulate IL-6 expression and *vice versa* in a feedback loop in melanoma cells. In the context of melanoma, WNT5A is an important player, whose increased expression could facilitate cell invasion and metastasis, thus promoting the progression of this disease [5, 19, 25, 26]. In support of our finding of a WNT5A-IL-6 positive feedback loop, both WNT5A [1, 5, 6] and IL-6 [7–10, 12] have been reported to promote tumour cell metastasis, although the role of IL-6 has not been completely explored in melanoma. However, an interesting study conducted by Hoejberg et al. demonstrated increased serum levels of IL-6 in melanoma patients, and these elevated levels of IL-6 could be linked to poor patient survival [8]. Further support for the existence of a WNT5A-IL-6 positive feedback loop comes from our analysis of a publically available online database [Heuristic Online Phenotype Prediction (HOPP) algorithm] [22]. In this analysis, we found elevated expression of both *WNT5A* and *IL-6* transcripts in invasive melanoma cells.

For the present study, we selected WM852 cells as an ideal system, first for their invasive nature (according to the HOPP database) and second for the increased levels of both WNT5A and IL-6. Although HTB63 and A375 cells are also stratified as invasive phenotypes (by HOPP), they express relatively low levels of WNT5A and IL-6 in comparison to the expression in WM852 cells. Using WM852 cells, we showed that either siRNA silencing of IL-6 or the neutralisation of endogenous IL-6 signalling could significantly reduce WNT5A expression in WM852 cells. In addition, siRNA silencing of WNT5A expression and the inhibition of endogenous WNT5A signalling using Box5 significantly reduced IL-6 secretion in WM852 melanoma cells. However, there was no reduction in *WNT5A* or *IL-6* mRNA levels following treatment with either an IL-6 neutralising antibody or Box5, which excludes the possibility that the WNT5A-IL-6 loop in WM852 cells is regulated at the transcriptional level.

Many examples of different positive feedback loops operating at the molecular/cellular levels that facilitate cancer cell invasion and metastasis exist. For example, in glioma cells, the pro-metastatic fibroblast growth factor-inducible 14 (Fn14) can induce its own expression through the activation of Rac1 and NF-κB, thereby promoting tumour cell migration and invasion [27]. Moreover, in breast cancer, invasive mesenchymal-like cancer cells can secrete GM-CSF, which stimulates the differentiation of macrophages into tumour-associated macrophages (TAMs). Reciprocally, these TAMs provide the cancer cells with increased amounts of the chemokine CCL18, which maintain the mesenchymal phenotype of the breast cancer cells, thus promoting breast cancer metastasis [28]. Although such mechanisms provide the tumours with an advantage in supporting cancer progression, they also widen the possibility of effective combinatorial treatment options to improve therapeutic efficacy. Considering the pro-metastatic role of both WNT5A and IL-6 in melanoma progression, a combined treatment that effectively inhibits the presently demonstrated WNT5A-IL-6 positive feedback loop is an attractive strategy to successfully impair melanoma cell invasion and metastasis.

Next, we investigated the efficacy of combined interference in the WNT5A-IL-6 positive feedback loop by inhibiting both WNT5A signalling and IL-6 signalling. As a proof of concept, we first demonstrated that combined siRNA knockdown of WNT5A and IL-6 more effectively inhibited the invasion of WM852 cells compared with the effects of individual siRNA knockdown of either WNT5A or IL-6. In the next set of experiments, we employed a more feasible therapeutic approach by combining Box5 (a WNT5A antagonist) and a neutralising anti-IL-6 antibody to investigate the efficacy of such a combined treatment on melanoma cell migration and invasion. Our results showed that the combined inhibition of WNT5A signalling (Box5) and IL-6 signalling (neutralising anti-IL-6 antibody) mimicked the results of the combined siRNA knockdown of WNT5A and IL-6 by more effectively reducing the migration and invasion of two different invasive melanoma cell lines, compared with the results from individual treatments with either Box5 or a neutralising anti-IL-6 antibody. These observations not only strengthen our demonstration of a WNT5A-IL-6 positive feedback loop but also, and more importantly, revealed the advantage of combined therapeutic inhibition as an attractive treatment strategy to prevent melanoma dissemination.

In summary, the present study demonstrates that WNT5A regulates its own expression in melanoma cells via a WNT5A-IL-6 positive feedback loop and that combined inhibition of both WNT5A signalling and IL-6 signalling could be an effective strategy to obstruct the dissemination of melanoma cells and thus slow or prevent disease progression.

## MATERIALS AND METHODS

### Microarray data analysis

The Zürich, Philadelphia, Mannheim, Wagner, Johansson and Augustine melanoma cell line microarray data sets were analysed online (http://www.jurmo.ch/hopp/hopp_mpse.php) using the Heuristic Online Phenotype Prediction (HOPP) algorithm. Retrieved normalised gene expression data of *WNT5A* (probe set 205990_s-at) and *IL*-6 (probe set 205207_at) from melanoma cell lines that scored as either proliferative or invasive by the HOPP algorithm were plotted and statistically analysed using Student's *t*-test. Melanoma phenotype signatures of WM852 (GSM109044), HTB63 (GSM162902) and A375 (GSM29663) cells were retrieved online (http://jurmo.ch/hopp/hopp_default_data.php) and presented as Widmer plots [22] (Figure S1).

### Cell culture and treatment

The WM852 human melanoma cell line originates from a metastatic site and its genetic background includes NRAS^Q61R^/BRAF^WT^/PTEN^+/−^/TP53^mutated^. The WM852 human melanoma cell line (Cat#WC00065) was procured in February 2015 from CORIELL Cell Repositories, Camden, NJ, USA. The identity of the WM852 cell line was confirmed by the supplier by short tandem repeat (STR) profiling using the AmpFlSTR^®^Identifiler^®^ PCR Amplification Kit (Cat#4322288) from Life Technologies, USA using loci consistent with all major worldwide STR standards. The HTB63 human melanoma cell line originates from a metastatic site and its genetic background includes BRAF^V600E^/NRAS^WT^/PTEN^−/−^/TP53^WT^. The A375 human melanoma cell line originates from a metastatic site and its genetic background includes BRAF^V600E^/NRAS^WT^/PTEN^WT^/TP53^WT^. Both the HTB63 human melanoma cell line (Cat#HT-144, LOT: 59550354) and the A375 human melanoma cell line (Cat#CRL-1619, LOT:61573377) were obtained from American Type Culture Collection (ATCC, VA, USA) in August 2014. The identities of the HTB63 and A375 cell lines were confirmed by ATCC by using multiplex PCR, which amplifies the amelogenin gene and eight of the most informative polymorphic markers in the human genome. The analysis of the amplicons was performed by Promega PowerPlex^®^ 1.2 system and the Applied Biosystems Genotyper 2.0 software. The WM852 cell line was purchased on the 23rd of February 2015, whereas the HTB63 and A375 cell lines were purchased on the 12th August 2014. Immediately upon arrival, all three cell lines were grown for 2 passages. Then, they were frozen in smaller aliquots that were thawed every fourth month as a fresh sample. Therefore, no cell line was used for more than 4 months.

All cell lines were regularly tested for the absence of mycoplasma contamination (EZ-PCR kit by Biological Industries, Bet Haemek, Israel) and grown as previously described. All cell lines were routinely serum starved prior to each experiment in 1% FBS-supplemented media for 24 h. For the neutralisation of secreted IL-6, required concentrations of mouse monoclonal anti-human IL-6 IgG_1_ antibody (Clone #6708, R&D Systems, Minneapolis, Minnesota, USA) were used and compared against equal concentrations of mouse IgG_1_ isotype control antibody (Clone #11711, R&D Systems, Minneapolis, Minnesota, USA). For the inhibition of WNT5A signalling, the indicated doses of Box5 (Calbiochem, San Diego, CA, USA) were used and compared to equal volumes of vehicle (NaHCO_3_ buffer, pH of approximately 7 or cell culture grade DMSO with a final concentration below 0.1%).

### Western blotting

Cells were lysed in 1 M Tris-HCl pH 7.5, 0.5 M NaCl, 30 mM sodium pyrophosphate, 50 mM sodium fluoride, 0.5 M EDTA, 1.5 mM MgCl_2_, 10% Glycerol, and 1% Triton X-100, supplemented with complete Mini EDTA-free protease inhibitor cocktail tablets (Roche Diagnostics, Indianapolis, Indiana, USA; 1 tablet/10 ml lysis buffer) and spun at 12,000 × *g* for 10 min at 4°C. Following the estimation of sample protein concentration using the Pierce^®^ BCA Protein Assay Kit (Thermo Scientific, Rockford, Illinois, USA), equal amounts of total protein (15 μg) were prepared in 4x Laemmli buffer and heated to 95°C for 5 min prior to loading on a SDS-PAGE gel. We used MDA-MB-468 breast cancer cell lysates as negative controls because they do not express endogenous WNT5A protein [12]. As positive controls, we used MDA-468 cell lysates supplemented with recombinant WNT5A protein (MDA-468 + rW5A). After the separation and transfer of the proteins to PVDF membranes, immuno-detection was performed using the following antibodies: primary antibodies, goat anti-WNT5A (1:100, R&D Systems, Minneapolis, Minnesota, USA) and mouse anti-α-tubulin (1:30000, Santa Cruz Biotechnology, Inc., Dallas, Texas, USA), secondary HRP-conjugated rabbit-anti-goat or goat anti-mouse antibodies (1:10000, Dako, Glostrup, Denmark). Chemiluminescent acquisitions of protein band intensities were obtained using the ChemiDoc^™^ imaging system (Bio-Rad Laboratories Inc., San Francisco, CA, USA) and densitometric quantification of relative protein expression was performed using Image Lab 3.0 software (Bio-Rad laboratories Inc., San Francisco, CA, USA).

### IL-6 ELISA

IL-6 release was measured in conditioned low serum cell culture media (only supplemented with 1% FBS) using the Human IL-6 ELISA Kit (Invitrogen, Carlsbad, CA, USA) according to the manufacturer's instructions. All experiments were conducted in 6-well plates, and collected cell culture supernatants were routinely centrifuged at 1000 × *g* for 5 min to remove cell debris and frozen at – 80°C prior to analysis. For the quantification of endogenous IL-6 release, plated WM852, HTB63 and A375 cells were maintained in cell culture media supplemented with 10% FBS for approximately 2 days until confluent. After reaching confluence, the cells were washed in PBS and 1.5 ml low serum media was added to each well. The cells were then allowed to condition the low serum cell culture media for 48 h. For analysis of IL-6 release following transient siRNA silencing of either IL-6 or WNT5A (72 h), treated cells were washed in PBS 24 h post-transfection and subsequently allowed to condition the low serum cell culture media for the remaining 48 h of each transient siRNA transfection. The amount of IL-6 release was then presented relative to negative control siRNA-transfected cells.

### RNA extraction, reverse transcriptase PCR and quantitative real-time PCR

Cells were washed twice with PBS, and total RNA was isolated using the RNeasy Plus Mini Kit (Qiagen, Hilden, Germany) according to the manufacturer's instructions. Equal amounts (1 μg) of RNA from each sample were used for cDNA synthesis using random primers and the M-MuLV reverse transcriptase enzyme (Thermo Scientific, Rockford, Illinois, USA). Quantification of the mRNA expression levels of *WNT5A, IL-6* and *IL-6R* and the endogenous TATA box binding protein (TBP) in the samples was carried out on a Stratagene Mx3005P system (Agilent Technologies, Santa Clara, CA, USA) using Maxima Probe/ROX QPCR Master Mix (Thermo Scientific) and primers, TaqMan Gene Expression Assays Hs01075666_m1 and Hs00427620_m1, respectively (Thermo Fisher Scientific, Waltham, MA, USA). For the relative quantification of *WNT5A, IL-6* and *IL-6R* levels, the comparative Ct method was performed using MxPro 4.10 software (Agilent Technologies, Santa Clara, CA, USA) and normalised against TBP.

### siRNA transfections

Transient siRNA transfections were performed as previously described [12] using the Lipofectamine 2000 transfection reagent (Invitrogen, Carlsbad, CA, USA) according to the manufacturer's instructions. Briefly, 400,000 cells/well seeded in 6-well plates were transfected with the indicated concentrations of the following siRNA oligonucleotides (all from Invitrogen): Negative Control (NC) siRNA (#4390843), anti-IL-6 siRNA #1 (s7311), anti-IL-6 siRNA #2 (s7312), anti-WNT5A siRNA #1 (s14871), and anti-WNT5A siRNA #2 (s14872).

### Cell invasion

Cell invasion assays were performed as previously described [12] using Matrigel pre-coated cell culture inserts (Corning, Bedford, MA, USA) with 8-μm-pore-size membranes and manually quantified by counting the number of invaded cells using an inverted light microscope (Nikon TMS, Japan). For experiments assessing the invasive capacity following either independent or combined siRNA silencing of WNT5A and IL-6, treated WM852 cells were allowed to invade during the last 24 h of each 72 h transient transfection. The results were presented as the relative invasion compared to negative control siRNA-transfected cells. To investigate how the inhibition of WNT5A signalling, the neutralisation of secreted IL-6 or a combination of both phenomena affected the invasive capacity of melanoma cells, WM852 and HTB63 cells were first pre-treated with Box5 (100 μM/ml) and/or anti-human IL-6 IgG_1_ antibody (1 μg/ml) alone or in combination for 24 h. After pre-treatment, invasion assays were performed over 24 h in the presence of the same dose of fresh Box5 and/or anti-human IL-6 IgG_1_ antibody. The results were presented as the relative invasion compared to cells treated with IgG_1_ isotype control antibody and vehicle (DMSO with a final concentration of 0.025%).

### Statistical analysis

Statistical analysis was carried out using GraphPad Prism 5.0 and IBM-SPSS software. Significant differences between two groups were determined by Student's *t*-tests, and those between multiple groups were determined using an analysis of variance (ANOVA). Multiple group analyses were checked for significance after an ANOVA using the Newman-Keuls multiple comparison test. The Pearson correlation test was used for correlation analysis of *WNT5A* and *IL-6* mRNA expression in melanoma cell/tissue based data sets. All the experiments were repeated in at least triplicate, and the data were expressed as the mean ± S.E.M. Differences were considered to be significant if the *p* value was less than 0.05.

## SUPPLEMENTARY MATERIALS FIGURES


