# Supplementary material for: Demonstration of a WNT5A-IL-6 positive feedback loop in melanoma cells: Dual interference of this loop more effectively impairs melanoma cell invasion
Source: Oncotarget. 2016 May 12;7(25):37790–802. doi: 10.18632/oncotarget.9332 (PMC5122349; doi:10.18632/oncotarget.9332)
Supplement: Supplementary file 1 [file oncotarget-07-37790-s001.pdf]

Demonstration of a WNT5A-IL-6 positive feedback loop in melanoma cells: Dual interference of this loop more effectively impairs melanoma cell invasion

Supplementary Materials

SUPPLEMENTARY MATERIALS AND METHODS

MTT cell viability assay

The MTT assay was performed to check the cytotoxicity of anti-human IL-6 IgG_1 antibody or Box5 in WM852 cells. Briefly, 15000 cells/well of WM852 cells were grown in 96-well flat-bottom tissue culture plates and treated with increasing concentrations of anti-human IL-6 IgG_1 antibody or Box5 for 48 h. In a separate treatment set, cells were exposed to fixed doses of either Box5 (100 μM) or anti-human IL-6 IgG_1 antibody (1 μg/ml) and their combination. For each treatment, cells exposed to the IgG_1 isotype control antibody or DMSO was considered to be the experimental control. After treatment, the media was removed and the wells were washed once with 1X PBS. Approximately 100 μL of 0.05% MTT reagent was added to each well followed by incubation at 37°C for 4 h to allow the formation of formazan crystals. Formazan crystals were dissolved, and colour intensity was measured spectrophotometrically at 570 nm.

Recombinant-IL-6 stimulation

To check for the presence of a WNT5A-IL-6 positive feedback loop, WM852 cells were first transiently transfected with *IL-6*-siRNA #1 for *IL-6* knockdown. The *IL-6* silenced cells were stimulated with recombinant-IL-6 for 48 h, and WNT-5A release was checked through western blotting.

Migration assay

Cell migration experiments were performed in both WM852 and HTB63 cells following 24 h pretreatment with individual or combination of Box5 or/and anti-human IL-6 IgG_1 antibody. After pretreatment, the cells were detached by versene application and suspended in 1% FBS- containing media. Approximately 50,000 cells/insert were seeded with fresh Box5 or/and anti-human IL-6 IgG_1 antibody (individually or in combination) treatment in the respective samples and allowed to migrate over a time period of 24 h. For quantification, the cells were fixed and stained with crystal violet. The quantification of cell migration was based on the cell numbers of each cell line, which was calculated either by counting the numbers of cells using NIH IMAGEJ© software or by spectroscopic methods.

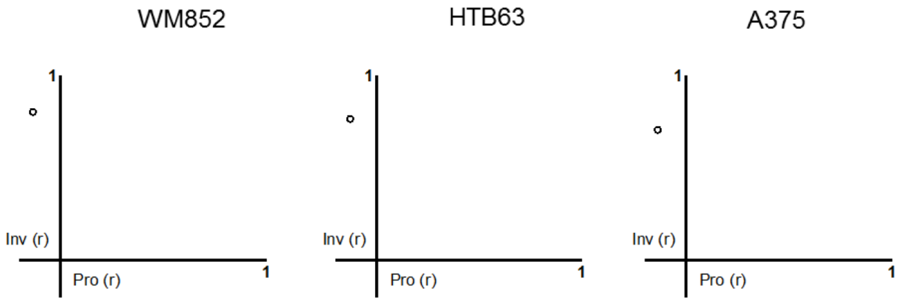

Accession ID	Cell Line	Pro (r)	Inv (r)
GSM109044	WM852	-0.1337	0.8041
GSM162902	HTB63	-0.1276	0.766
GSM29663	A375	-0.1379	0.7073

Supplementary Figure S1: HOPP phenotype signature of WM852, HTB63 and A375 cells. Widmer plots showing the phenotype signatures of the human melanoma cell lines WM852, HTB63 and A375 as analysed by the Heuristic Online Phenotype Prediction (HOPP) algorithm.

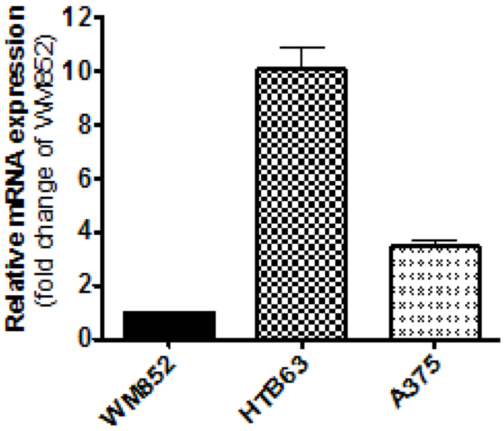

Supplementary Figure S2: IL-6R mRNA expression in WM852, HTB63 and A375 cells. QPCR analysis of endogenous *IL-6R* mRNA expression levels in human WM852, HTB63, and A375 cells. Samples were normalised against *TATA-binding protein (TBP)* mRNA expression, and the results are presented relative to the *IL-6R* mRNA expression of WM852 cells.

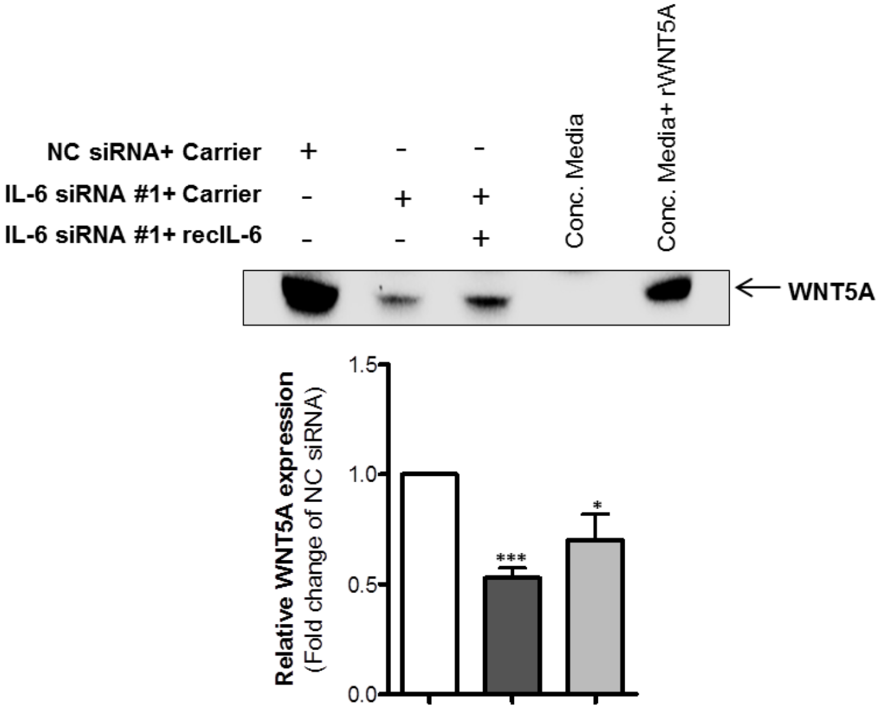

Supplementary Figure S3: Recombinant IL-6 stimulation recovers WNT5A release in IL-6-silenced WM852 cells. The release of WNT5A in WM852 cells post-*IL-6*-siRNA transfection was evaluated through immunoblotting. Briefly, equal volumes of the treatment medium were collected from NC siRNA, *IL-6*-siRNA #1 transfected and recombinant IL-6-stimulated (48 h) WM852 cells, and WNT5A release was determined in the concentrated sample. Recombinant WNT5A was used as a positive control for WNT5A detection in the blots.

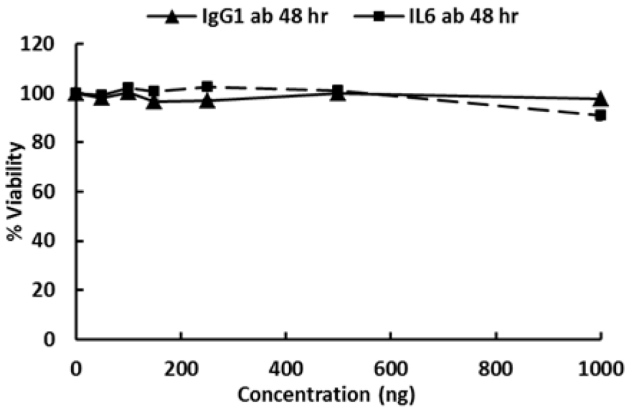

Supplementary Figure S4: Effect of IL-6 neutralising antibody on the viability of WM852 cells. An MTT cell viability assay was performed after WM852 cells were exposed to increasing doses of human anti-IL-6 antibody for 48 h as described in the Supplementary Materials and Methods section. The results were evaluated at 570 nm using a multi-well plate reader.

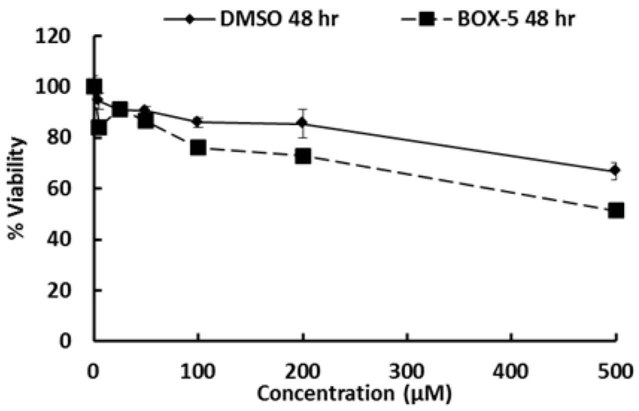

Supplementary Figure S5: Effect of the WNT5A-derived antagonistic peptide Box5 on WM852 cell viability. An MTT cell viability assay was performed after WM852 cells were exposed to increasing doses of human Box5 for 48 h as described in the Supplementary Materials and Methods section. Readings were taken at 570 nm using a multi-well plate reader.

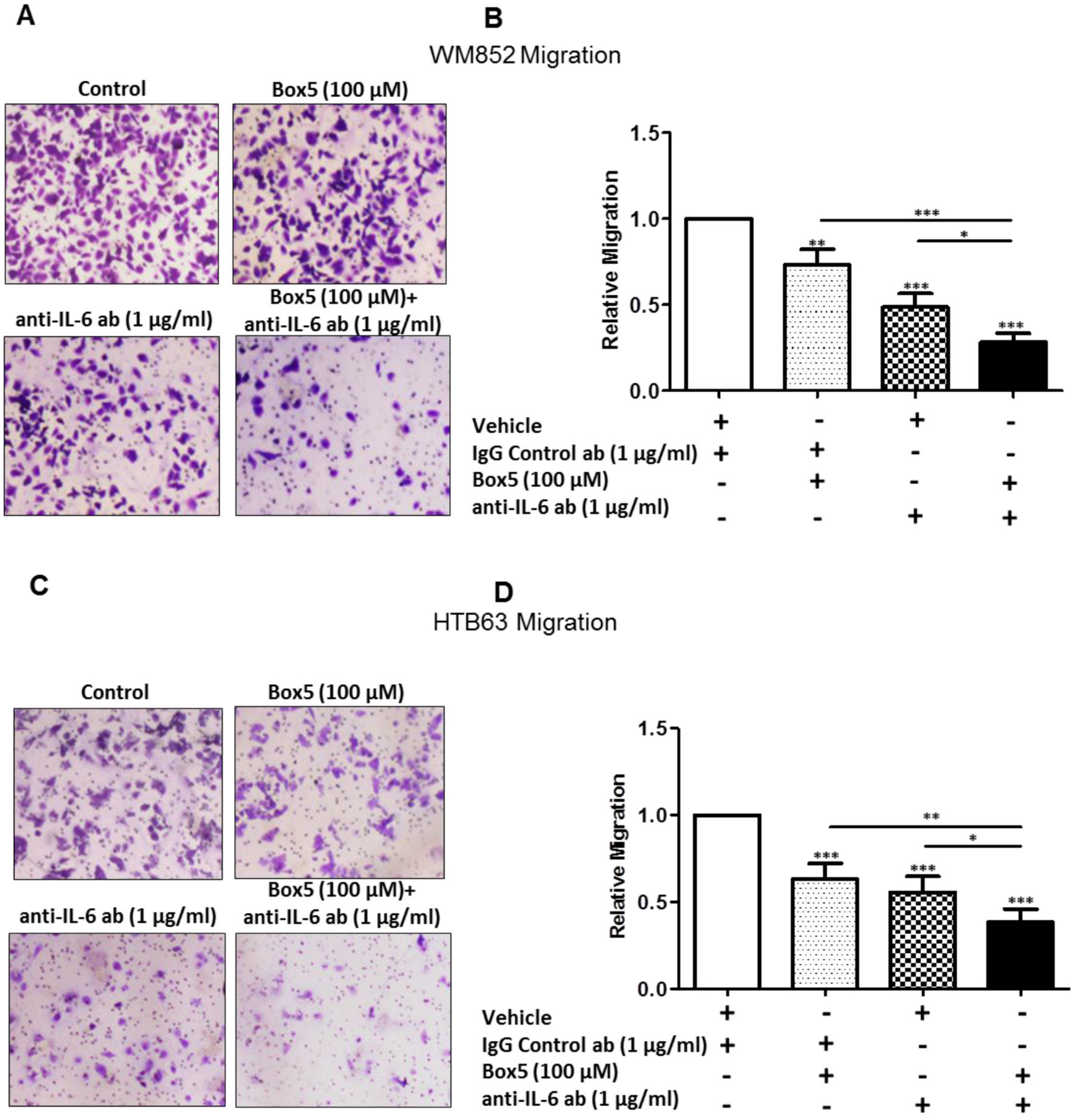

Supplementary Figure S6: The combination of Box5 and anti-IL-6 antibody more effectively impairs WM852 and HTB63 cell migration. (**A**) transwell-based migration assay was performed to evaluate the cell migration efficiency of (**A** and **B**) WM852 and (**C** and **D**) HTB63 treated with either Box5 and neutralising anti-IL-6-antibody alone or their combination as described in the Supplementary Materials and Methods section. Proper controls (DMSO for Box5 treatment or IgG_1 Isotype antibody for neutralising anti-IL-6-antibody treatment) were used to compare the effect of the treatments on the cell migration. The results are given as means and S.E.Ms; $^*p < 0.05$; $^{**}p < 0.01$; $^{***}p < 0.001$.

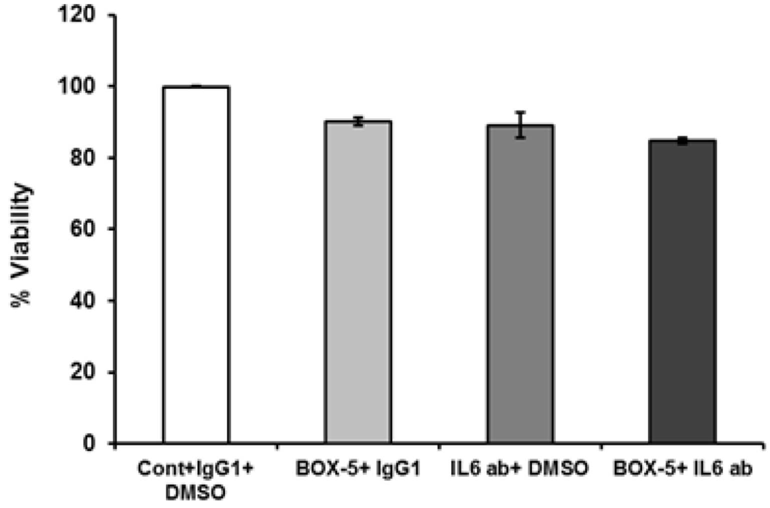

Supplementary Figure S7: Combinatorial effect of Box5 and anti-IL-6-antibody on WM852 cell viability. WM852 cells were exposed to either individual Box5 or anti-IL-6 antibody for 48 h as described in the Supplementary Materials and Methods section. Readings were taken at 570 nm using a multi-well plate reader.
